# Novel Protein-Protein Inhibitor Based Approach to Control Plant Ethylene Responses: Synthetic Peptides for Ripening Control

**DOI:** 10.3389/fpls.2017.01528

**Published:** 2017-09-05

**Authors:** Mareike Kessenbrock, Simone M. Klein, Lena Müller, Mauricio Hunsche, Georg Noga, Georg Groth

**Affiliations:** ^1^Institute of Biochemical Plant Physiology, Heinrich Heine University Düsseldorf Düsseldorf, Germany; ^2^Institute of Crop Science and Resource Conservation – Horticultural Science, University of Bonn Bonn, Germany; ^3^COMPO EXPERT GmbH Münster, Germany; ^4^Bioeconomy Science Center, Forschungszentrum Jülich Jülich, Germany

**Keywords:** ethylene signaling, ethylene receptors, peptide, ripening control, *Solanum lycopersicum* (tomato), post-harvest application

## Abstract

Ethylene signaling is decisive for many plant developmental processes. Among these, control of senescence, abscission and fruit ripening are of fundamental relevance for global agriculture. Consequently, detailed knowledge of the signaling network along with the molecular processes of signal perception and transfer are expected to have high impact on future food production and agriculture. Recent advances in ethylene research have demonstrated that signaling of the plant hormone critically depends on the interaction of the ethylene receptor family with the NRAMP-like membrane protein ETHYLENE INSENSITIVE 2 (EIN2) at the ER membrane, phosphorylation-dependent proteolytic processing of ER-localized EIN2 and subsequent translocation of the cleaved EIN2 C-terminal polypeptide (EIN2-CEND) to the nucleus. EIN2 nuclear transport, but also interaction with the receptors sensing the ethylene signal, both, depend on a nuclear localization signal (NLS) located at the EIN2 C-terminus. Loss of the tight interaction between receptors and EIN2 affects ethylene signaling and impairs plant ethylene responses. Synthetic peptides derived from the NLS sequence interfere with the EIN2–receptor interaction and have utility in controlling plant ethylene responses such as ripening. Here, we report that a synthetic peptide (NOP-1) corresponding to the NLS motif of *Arabidopsis* EIN2 (aa 1262–1269) efficiently binds to tomato ethylene receptors LeETR4 and NR and delays ripening in the post-harvest phase when applied to the surface of sampled green fruits pre-harvest. In particular, degradation of chlorophylls was delayed by several days, as monitored by optical sensors and confirmed by analytical methods. Similarly, accumulation of β-carotene and lycopene in the fruit pulp after NOP-1 application was delayed, without having impact on the total pigment concentration in the completely ripe fruits. Likewise, the peptide had no negative effects on fruit quality. Our molecular and phenotypic studies reveal that peptide biologicals could contribute to the development of a novel family of ripening inhibitors and innovative ripening control in climacteric fruit.

## Introduction

Worldwide, a tremendous amount of food produced for human consumption is lost or wasted until the product reaches the consumer, with about 50% of those food losses being valuable vegetables and fruits (Blanke, 2014). The tomato fruit is one of the most important climacteric fruits (Jackman et al., 1990; Vidoz et al., 2010) and has worldwide high economic and nutritional importance, mainly because of its high concentrations of carotenoids such as lycopene, β-carotene and pro-vitamin A (Canene-Adams et al., 2005; Vidoz et al., 2010) which accumulate during fruit ripening. The ripening process of climacteric fruits is characterized by a strong increase in cell respiration which is mainly regulated by the plant hormone ethylene (Alexander and Grierson, 2002; Guo and Ecker, 2004). Ripening is initiated by a burst of an auto-stimulated ethylene synthesis, with following activation of ripening related genes (Abano and Buah, 2015). This ethylene related gene expression leads to physiological, morphological and biochemical changes. In the process of fruit ripening, fruits change color, texture, firmness, flavor and aroma (Brady, 1987; Alexander and Grierson, 2002) due to degradation of pectins, cellulose and chlorophyll as well as due to a decreasing content of organic acids and increasing concentration of soluble sugars, carotenes and aroma volatiles (Brady, 1987).

Besides the traditional quality analysis of firmness and content of sugars, acids, vitamins and pigments, changes in fruit ripening and fruit quality might be evaluated by non-destructive optical methods (Abbott, 1999; Hoffmann et al., 2015). Analogous to that, consumers usually estimate fruit quality based on fruit skin color. Color development of tomatoes from green to red can be measured by monitoring chlorophyll degradation as well as lycopene and β-carotene accumulation (Alexander and Grierson, 2002). Typically, the total content of these pigments is analyzed using wet chemical procedures (Barros et al., 2007; Azeez et al., 2012; Kalogeropoulos et al., 2012), whereas non-destructive optical sensors that evaluate overall changes in fruit color based on reflection and fluorescence properties provide monitoring parameters that strongly correlate with the analytical values (McGuire, 1992; Hoffmann et al., 2015).

Control of ripening is important to ensure quality and to reduce post-harvest losses of climacteric fruits. At commercial scales, fruits are usually stored at low temperatures under controlled atmosphere to limit ethylene production and ethylene response (Watkins et al., 2000; Saltveit, 2005; Passam et al., 2007). Alternative approaches, such as genetic engineering of ethylene biosynthesis to decrease endogenous ethylene production, are under development in science and research despite of ongoing discussion in Europe about genetic engineering in general. At the production scale, fruit maturation can be delayed with aminoethoxyvinylglycine (AVG), an inhibitor of ACC-synthase, the key enzyme of ethylene biosynthesis (Saltveit, 2005). For post-harvest treatment, 1-methylcyclopropene (1-MCP), a gaseous chemical with the ability to inhibit ethylene receptors and receptor-triggered ethylene response can be applied (Watkins et al., 2000; Yuan and Carbaugh, 2007).

Pathways and mechanisms for biosynthesis, perception and signal transduction of the plant hormone ethylene have been extensively studied in the model plant *Arabidopsis thaliana*. These studies disclosed that the ethylene signal is perceived by a family of five receptor proteins, which form homo- and heterodimers at the ER membrane and function as negative regulators of the ethylene response (Bleecker et al., 1988; Chang et al., 1993; Hua et al., 1995, 1998; Hua and Meyerowitz, 1998; Grefen et al., 2008). Although the exact output of the receptors is still obscure, genetic studies demonstrate that in the absence of ethylene, receptors activate the Raf-like protein kinase CONSTITUTIVE TRIPLE RESPONSE 1 (CTR1), another negative regulator of the pathway (Kieber et al., 1993). Downstream of the receptors and the ER associated CTR1 kinase the membrane protein ETHYLENE INSENSITIVE 2 (EIN2), which contains a highly conserved NLS (Bisson and Groth, 2011; Qiao et al., 2012) shown to mediate interaction with the up-stream receptors (Bisson and Groth, 2015; Bisson et al., 2016), implements a positive regulatory role on ethylene signaling. In the presence of ethylene, the receptors bind the hormone and become inactivated. CTR1 cannot be activated by the receptors, and the lack of CTR1 activation cannot phosphorylate EIN2. Subsequently, the C-terminal end of EIN2 (C-END) containing the NLS-motif is cleaved off by an unknown mechanism and translocated to the nucleus (Ju et al., 2012; Qiao et al., 2012; Wen et al., 2012). In the nucleus, the EIN2 C-terminus directly or indirectly stabilizes the transcription factor EIN3 (Wen et al., 2012; Li et al., 2015) and its paralogous, the EIN3-like proteins (EILs), and transcription of ethylene response genes is activated (Chao et al., 1997; Solano et al., 1998).

In analogy to the model plant *Arabidopsis*, tomato contains a multigene family of the ethylene receptors. In total, seven isoforms named LeETR1, LeETR2, NR, LeETR4, LeETR5, LeETR6, and LeETR7 have been identified (Wilkinson et al., 1995; Zhou et al., 1996a,b; Lashbrook et al., 1998; Tieman and Klee, 1999) which are structurally diverse sharing at the most extreme less than 50% sequence identity. Similar to their *Arabidopsis* relatives the tomato receptors cluster in two subfamilies. LeETR1, LeETR2 and NR forming the subfamily I are characterized by a functional histidine kinase domain and a sensor domain consisting of three transmembrane helices. An additional putative membrane-spanning domain is present in LeETR5, LeETR6, LeETR7, and possibly in LeETR4 of subfamily II which are further characterized by a degenerated histidine kinase domain. All receptors except for NR contain a C-terminal response regulator domain (Lashbrook et al., 1998; Tieman and Klee, 1999). Expression patterns vary among the different receptor isoforms. While LeETR1 is expressed constitutively in all tissues, expression of LeETR2 is bound to seed germination and leaf senescence. NR, LeETR4 and to a lower extent LeETR5 are found at high expression levels in ripening fruit (Payton et al., 1996; Lashbrook et al., 1998; Tieman and Klee, 1999), but are rapidly degraded in the presence of ethylene by a 26S proteasome dependent pathway (Kevany et al., 2007). Due to this strong post-translational regulation of their protein level by the plant hormone and the observed correlation of receptor content and fruit ripening (Kevany et al., 2007), these receptors are of particular interest for studying the molecular effect of ripening inhibitors targeting ethylene signaling.

Recent insights in the ethylene signaling pathway propose a novel way to interfere with fruit ripening based on a yet unknown function of the NLS in the ethylene signaling protein EIN2. Peptides such as the synthetic octapeptide LKRYKRRL (NOP-1) mimicking this NLS motif were shown to block the interaction of EIN2 and ETR1 receptors and reduce plant ethylene responses (Bisson and Groth, 2015; Bisson et al., 2016).

In this study, we demonstrate that the NOP-1 octapeptide also efficiently binds to the ripening related tomato receptors NR and LeETR4 structurally divergent from ETR1. Moreover, we provide quantitative measures of the ripening delay related to NOP-1 treatment such as pigment content, overall color analysis and fruit firmness. Our data show that surface application of NOP-1 on tomato fruits can delay ripening without impairment of fruit quality.

## Materials and Methods

### Cloning of Tomato Receptors LeETR4 and NR into Expression Vector pET16b

Full-length codon optimized cDNA sequences encoding tomato ethylene receptors LeETR4 and NR (UniProt ID: LeETR4 Q9XET8; NR Q41341) were ordered at GenScript United States according to published sequences (NCBI ID: LeETR4 NM_001247276.2; NR NM_001246965.2). Construction of expression vector pET16b (Novagen, Madison, WI, United States) carrying the target DNA sequence, an ampicillin resistance and a deca-histidine tag were performed by Gibson Assembly (Gibson et al., 2009). For amplification of linearized vector forward primer 5′-GGATCCGGCTGCTAACAAAGC-3′ and reverse primer 5′-ATGACGACCTTCGATATGGC-3′ were used. LeETR4 was amplified using forward primer 5′-ATCGAAGGTCGTCATATGCTGCGTACCCTGGCGAG-3′ and reverse primer sequence 5′-TTAGCAGCCGCCTTACATCAGAGCTGGATTACGGCTACCACGCA-3′. For amplification of NR forward primer 5′-ATCGAAGGTCGTCATATGGACGATTGCATT-3′ and reverse primer 5′-TTAGCAGCCGGATCCTTACAGGCTACGCTGATAACGCT-3′ were used. Amplified fragments were added to Gibson Assembly Master Mix containing an exonuclease, a DNA polymerase and a ligase to assemble a circular plasmid with LeETR4 and NR coding sequence, respectively. Reaction assays were incubated at 50°C for 10 min and at 40°C for 60 min. Assembled plasmids were transformed into *E. coli* strain XL 1-blue and sequenced by Seqlab (Göttingen, Germany) to verify correctness.

### Expression of Recombinant Tomato Receptors LeETR4 and NR in *E. coli*

For expression of recombinant LeETR4 and NR the related pET16b expression vectors were transformed into *E. coli* strains C43 and BL21 (DE3), respectively. Cells were grown in 2YT medium [1.6% (w/v) peptone, 1% (w/v) yeast extract and 0.5% (w/v) NaCl] with 2% ethanol and 100 μg/mL ampicillin at 30°C. At OD_600_ = 0.4 temperature was reduced to 16°C. Expression of tomato receptors was induced at OD_600_ = 0.6 by the addition of 0.5 mM isopropyl-β-d-1-thiogalactopyranoside (IPTG). Cells were grown and harvested after 20 h (LeETR4) or 6 h (NR) by centrifugation for 15 min at 7,000 × *g* and 4°C. Expression of tomato receptors was analyzed by SDS–PAGE (Laemmli, 1970) and detected by Western blotting (Towbin et al., 1979).

### Solubilization and Purification of Recombinant Tomato Receptors LeETR4 and NR

The resulting cell pellet after expression was resuspended in PBS pH 8, 10% (w/v) glycerol, 1 mM dithiothreitol and 0.002% (w/v) phenylmethylsulfonyl fluoride (PMSF). DNase I (10 μg/mL) was added before cells were broken with Constants Cell Disruption System (Constant Systems, Daventry, United Kingdom) at 2.4 kbar and 5°C. Cell lysate was centrifuged for 30 min at 14,000 × *g* and 4°C. The resulting supernatant was centrifuged again for 30 min at 40,000 × *g* and 4°C. The pellet was resuspended in PBS buffer and centrifuged for 30 min at 34,000 × *g* and 4°C. For solubilization the pellet was resuspended in 50 mM Tris/HCl pH 8, 200 mM NaCl, 1.2% (w/v) FosCholine-16, 0.002% (w/v) PMSF (buffer S) and stirred at RT and 700 rpm for 1 h. Membrane fragments were isolated by ultracentrifugation (229,600 × *g*, 4°C, 30 min). The supernatant was loaded to a 5 mL HisTrap FF column operated by an ÄKTAprime plus (both GE Healthcare Life Sciences) at 4°C equilibrated with buffer A [buffer S containing 0.015% (w/v) FosCholine-16], followed by an ATP washing step of 20 column volumes [50 mM Tris/HCl pH 8, 200 mM NaCl, 50 mM KCl, 20 mM MgCl_2_, 10 mM ATP and 0.002% (w/v) PMSF]. The column was washed with 50 mM imidazole and receptors eluted with 250 mM imidazole. Purified proteins were concentrated to 2.5 mL and buffer was changed to 100 mM potassium phosphate buffer pH 7.3, 300 mM NaCl, 0.015% (w/v) FosCholine-16, 0.002% (w/v) PMSF for labeling with Alexa Fluor 488-Maleimide (Thermo Fisher Scientific) on a PD-10 column (GE Healthcare Life Sciences). Alexa Fluor 488-Maleimide was applied to the protein in 2.5-fold excess and incubated for 30 min at RT. Then, buffer was changed to 50 mM Tris/HCl pH 8, 300 mM NaCl, 5% (w/v) glycerol, 0.015% (w/v) FosCholine-16, 0.002% (w/v) PMSF. Purity of LeETR4 and NR was analyzed by SDS–PAGE (Laemmli, 1970) with colloidal Coomassie staining (Dyballa and Metzger, 2009) and Western blotting (Towbin et al., 1979) using a directly conjugated Anti-His-HRP monoclonal antibody (Miltenyi Biotech, Bergisch Gladbach, Germany). Proper folding of receptors was verified by CD-spectroscopy (Classen and Groth, 2012; Kessenbrock and Groth, 2017).

### CD Spectroscopy of Recombinant Tomato Receptors

CD measurements were performed in a Jasco J715 spectropolarimeter (Jasco GmbH, Gross-Umstadt, Germany). For the far UV spectra a cylindrical quartz cuvette from Hellma Analytics (Muellheim, Germany) with 1-mm-path-length was used. Purified tomato receptors LeETR4 and NR were dissolved to a final concentration of 0.2 mg ml^-1^ in 10 mM potassium phosphate pH 8.0 and 0.0075% (w/v) FosCholine-16. Protein and FosCholine-16 concentrations were determined by a Direct Detect Infrared Spectrometer (Merck Chemicals GmbH, Darmstadt, Germany) (Strug et al., 2014). For detailed information on protein preparation see Kessenbrock and Groth (2017). Measurements were run at ambient temperature. Each protein sample was recorded in the range of 260–185 nm. The CD spectra were obtained by averaging ten individual spectra using a bandwidth of 1 nm at 50 nm min^-1^. Secondary structure content of purified proteins were calculated from the spectra by CDSSTR and CONTINLL (Provencher and Gloeckner, 1981; Johnson, 1999).

### Binding Studies of NOP-1 at Tomato Receptors LeETR4 and NR by Microscale Thermophoresis

Binding of the NOP-1 octapeptide to purified recombinant tomato receptors LeETR4 and NR was analyzed by microscale thermophoresis (MST) (Duhr and Braun, 2006; Wienken et al., 2010; Jerabek-Willemsen et al., 2011; Seidel et al., 2013). Receptors (100 nM) labeled with Alexa Fluor 488-Maleimide (Thermo Fisher Scientific) were titrated with peptide ligand NOP-1 dissolved in 50 mM Tris–HCl pH 8.0, 300 mM NaCl at concentrations from 500 μM to 61.04 nM. Then samples were transferred into standard glass capillaries and thermophoresis was measured using a Monolith NT.115 (NanoTemper Technologies GmbH, München, Germany). MST measurements were recorded at 20% MST power for LeETR4-NOP-1 and 60% MST power for NR-NOP-1, respectively. Receptors were chemically denatured by incubation with the strong ionic detergent SDS [4% (w/v)] and the small-molecule redox reagent DTT (40 mM) for 5 min in the dark at RT and served as control to confirm specific and selective binding of the ligand. All measurements were run in triplicate.

### Fruit Material, Treatments and Storage Conditions

Tomato fruits (*Solanum lycopersicum* L.) of the cultivar ‘Lyterno’ (Rijk Zwaan, De Lier, Netherlands) were harvested at the maturity stage “green” (USDA; 2005) from tomato plants which where cultivated in a commercial-like greenhouse at the research station Campus Klein-Altendorf (University of Bonn, Germany). At the early development stages, the trusses were manually thinned to six fruits per truss, according to the common practice aiming standardized fruit size and quality. For the experiment, the last two fruits of the fourth truss, counted from the bottom, were chosen for evaluations. At the beginning of the experiment, 150 fruits showing similar color and size were divided into four treatments (*n* = 25 fruits per treatment). On each fruit, a transparent polyethylene film was placed at the equatorial zone to demark the four evaluation points of 2.8 cm diameter each.

The four treatments were as follows: (1) control; (2) NOP-1, 400 μM; (3) NOP-1, 1000 μM; (4) NOP-1, 2000 μM. The NOP-1 peptide (GenScript, Piscataway, NJ, United States) was dissolved in 5 ml deionized water. On each fruit, a total of 200 microdroplets (0.5 μL each) of the peptide solution were gently deposited (50 micro droplets on each marked area of the tomato fruit) with a Hamilton microdispenser (Hamilton Bonaduz AG, Bonaduz, Switzerland). Fruits of the control treatment received an equal number of microdroplets of deionized water. After application of the droplets fruits of each treatment were allocated in storage boxes thereby avoiding fruit-to-fruit contact, and stored at room temperature (19 ± 2°C).

### Non-destructive Measurements of Fruit Color at Ripening

Starting at the beginning of the experiment until 28 days after treatments (DAT), fruit ripening was evaluated and monitored twice a week with two non-destructive sensors using the principles of light reflectance and fluorescence emission. Evaluations were done on the marked fruit zones (*n* = 4 areas/fruit, *n* = 25 fruits per treatment).

Changes of the surface color over time were determined with a portable spectrophotometer (CM-503d, Konica Minolta Inc., Tokyo, Japan), which has a sensing area of 7 mm^2^. Based on the CIELAB model (McGuire, 1992), the recorded parameters are converted into the hue° index. Hue° was calculated according to the following formula:

$${\mathrm{hue}}^{{}^{\circ}}=\tan^{-1}\left(\frac{b^{*}}{a^{*}}\right),$$

where ‘*a^∗^*’ and ‘*b^∗^*’ are defined as color coordinates, provided that ‘*a^∗^*’ is the point of the green-red axis and ‘*b^∗^*’ is defined as the point of the yellow-blue axis. On the basis of the above, the resulting angle is converted into the corresponding color value.

### Fluorescence Based Analysis of Fruit Maturity

Pigment fluorescence was applied as second non-destructive technique to address fruit maturation. To this end, a handheld device (Multiplex^®^3, Force-A, Orsay, France) equipped with light-emitting diodes (LEDs) with UV (375 nm), blue (475 nm), green (510 nm) and red (635 nm) excitation was used. Fluorescence was detected in the blue-green (BGF, 425–475 nm), red (RF, 680–690 nm) and near-infrared (FRF, 720–755 nm) spectral regions (Ben Ghozlen et al., 2010). Based on the absolute fluorescence signals recorded in a detection diameter of approximately 2 cm, simple and complex fluorescence ratios were calculated. The parameter Simple Fluorescence Ratio excited with red light (SFR_R) as a measure of the chlorophyll content is as follows:

SFR_R = (FRF_R/RF_R) (according to Ben Ghozlen et al., 2010).

### Determination of Chlorophyll, β-Carotene and Lycopene

Determination of the pigment content in the fruits was done weekly on five fruits each treatment. The concentrations of β-carotene, lycopene and chlorophyll were analyzed from freeze-dried and ground fruit samples, as described below. Concentrations of chlorophyll, β-carotene and lycopene were analyzed according to the method of Nagata and Yamashita (1992) as described by Barros et al. (2007), Azeez et al. (2012) and Kalogeropoulos et al. (2012) with the following modifications. Briefly, 1.5 ml of the solvent Aceton:Hexane (4:6) was added to 0.1 g of the freeze-dried and ground material, homogenized and centrifuged for 10 min at 16,100 × *g* (CENTRIFUGE 5415 R, Eppendorf AG, Hamburg, Germany). Next, 2 ml of the solvent was added to the supernatant and the absorption of the solutions was determined at 435, 505, 645, and 663 nm in a LAMBDA 35 spectrophotometer (PerkinElmer^®^, Waltham, MA, United States). Concentrations of chlorophyll, β-carotene and lycopene were calculated according to Nagata and Yamashita (1992) by the following equations:

chlorophyll a[mg/100 ml]=0.999A663-0.0989A645chlorophyll b[mg/100 ml]=-0.328A663+1.77A645β-carotene[mg/100 ml]=0.216A663-0.304A505-0.452A453lycopene[mg/100 ml]=-0.0458A663+0.204A645+0.372A505-0.0806A453

Total chlorophyll was calculated by adding chlorophyll a and chlorophyll b content.

### Statistical Analysis

All results are expressed as mean ± SE. Analyses of variance were determined with one-way ANOVA (α ≤ 0.05). In case of statistical significance, the Tukey’s HSD (α ≤ 0.05) was applied to establish the differences among means. Statistical analyses were carried out using SPSS 22.0.

## Results

### Expression and Purification of Tomato Receptors LeETR4 and NR

**FIGURE 1:**
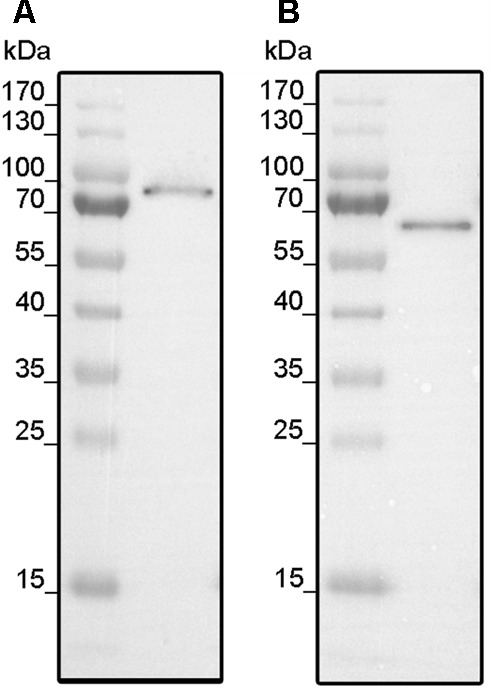
Expression of LeETR4 **(A)** and NR **(B)**. **(A)** *E. coli* C43 (DE3) was transformed with pET16b_LeETR4 and expressed for 20 h after induction at 16°C. **(B)** pET16b_NR was transformed into *E. coli* BL21 (DE3) and expressed for 6 h after induction at 16°C. For both expressions, host cell extract was analyzed by Western blotting using an Anti-His antibody targeting deca-histidine tagged proteins.

Codon optimized synthetic genes encoding full-length ethylene receptors LeETR4 and NR were each cloned into expression vector pET16b by Gibson Assembly Cloning (Gibson et al., 2009). Expression vectors encoding the tomato receptors were transformed into cells of *E. coli* strains BL21 (DE3) and C43 (DE3) which have been successfully applied for the expression of different members of the ethylene receptor family from *A. thaliana, Lycopersicon esculentum* and *Physcomitrella patens* in previous studies (Voet-van-Vormizeele and Groth, 2008; Classen and Groth, 2012). Protein expression was induced by the addition of 0.5 mM IPTG. Optimum expression was obtained for LeETR4 after 20 h in C43 (DE3) at 16°C, while systematic analysis of expression parameter for NR showed best expression after 6 h in BL21 (DE3) at 16°C (**Figure 1**).

**FIGURE 2:**
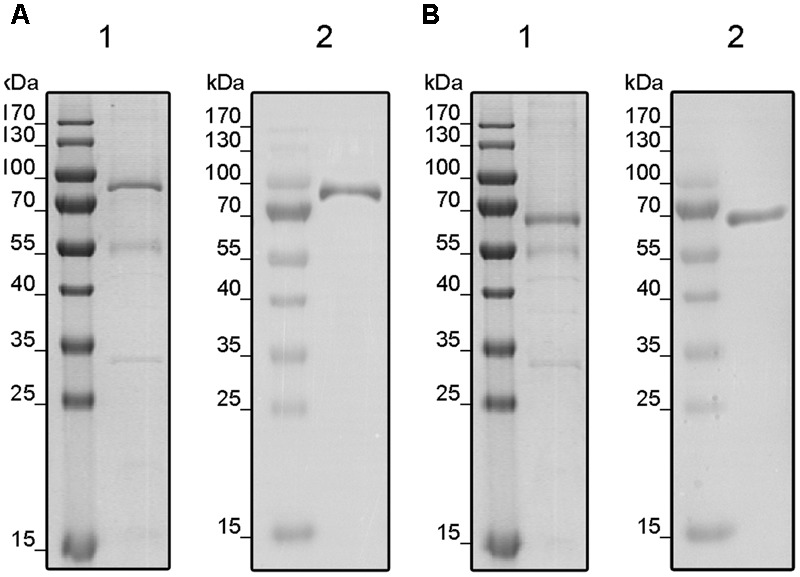
Purification of solubilized and His-tagged LeETR4 **(A)** and NR **(B)** by IMAC. Purified proteins LeETR4 and NR were analyzed by SDS-PAGE. Purified proteins were visualized by colloidal Coomassie staining (1) and Western blotting (2) using an anti-His antibody.

Receptors were localized in the membrane fractions of the host and solubilized from these membranes by the mild detergent Fos-Choline-16. After solubilization, receptors were purified in a single chromatography step on Ni–NTA agarose (GE Healthcare Life Sciences, Munich, Germany). Purification of the recombinant tomato receptors was analyzed by SDS–PAGE. The related protein gels (**Figure 2**) show prominent bands at 90 and 70 kDa corresponding to the molecular weight of LeETR4 (88 kDa) and NR (74 kDa). Besides, two minor contaminations were detected in the lower MW range at 55 and 32 kDa, respectively. Hence, both receptors have been successfully purified from their heterologous host. Identity of the receptors was confirmed by antibodies directed against the deca-histidine tag in both proteins.

### Secondary Protein Structure and Functional Folding of Purified LeETR4 and NR

**FIGURE 3:**
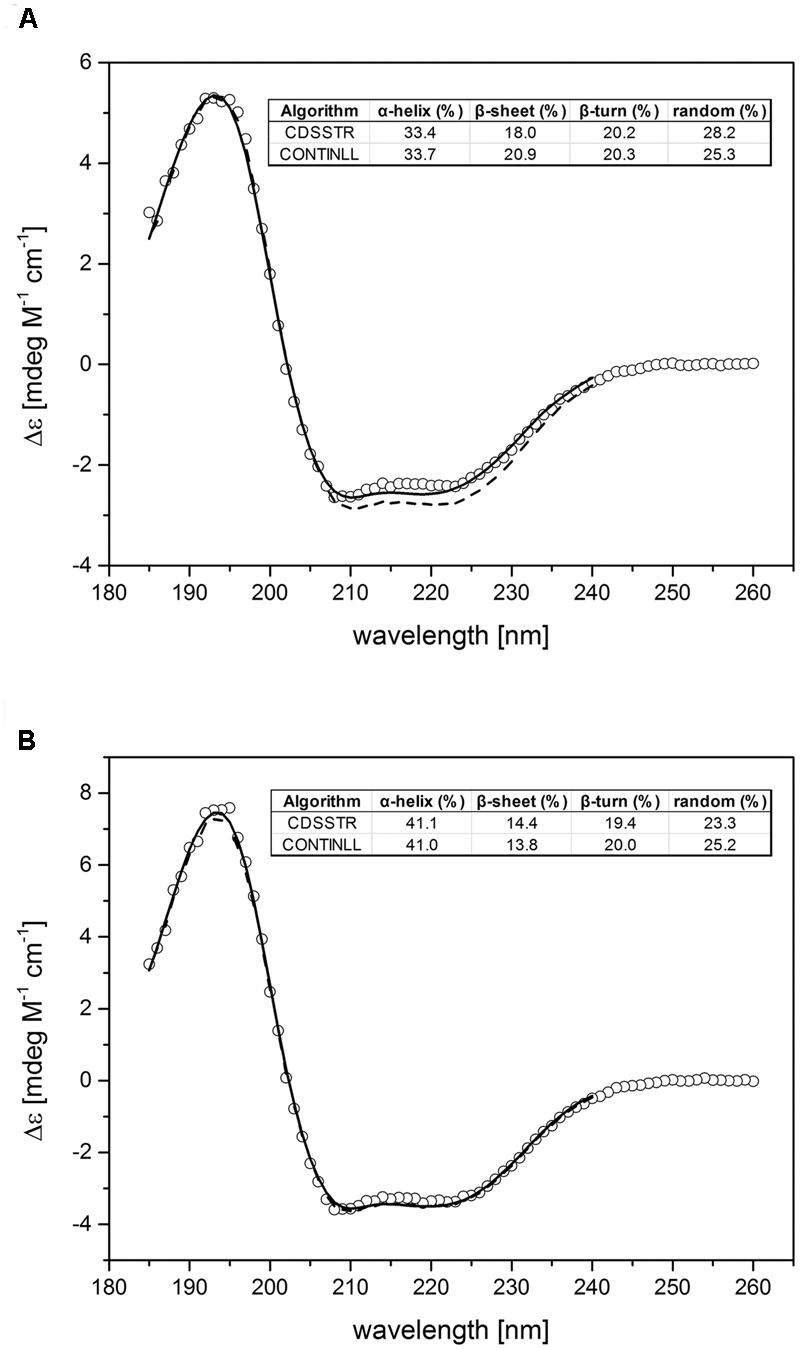
Experimental and calculated CD spectra of purified LeETR4 **(A)** and NR **(B)**. The far-UV CD spectra of LeETR4 and NR (○) were obtained by accumulating 10 spectra with 1 nm bandwidth and a scanning speed of 50 nm min^-1^. The CD data were adjusted to molar extinction (Δ𝜀) considering molecular weight and protein concentration of the ethylene receptors. The secondary structure calculations were determined by using the CDSSTR (dashed line) and CONTINLL (solid line) program (Provencher and Gloeckner, 1981; Johnson, 1999).

Folding and protein secondary structure of purified recombinant tomato receptors were probed by CD spectroscopy. The corresponding spectra of LeETR4 and NR shown in **Figure 3** are typical of partially helical proteins, displaying two minima at approximately 209 and 222 nm with an isosbestic point at 202 nm. Overall, the spectra of both receptor proteins are highly similar and correspond to previous CD data on receptor orthologs from *Arabidopsis* and *Physcomitrella* (Classen and Groth, 2012). Secondary structure calculations by CDSSTR and CONTINLL suggest an α-helix content of 34% and a β-sheet percentage of 18–20% for LeETR4. Similar numbers of 41–42% α-helix and 14% β-sheet structure were obtained for NR. Consequently, CD spectroscopic measurements verify that the purified receptors adopt a well-folded structure and are indicative for a native conformation of the recombinant tomato proteins.

### Binding of NOP-1 to Purified Recombinant Tomato Receptors LeETR4 and NR

**FIGURE 4:**
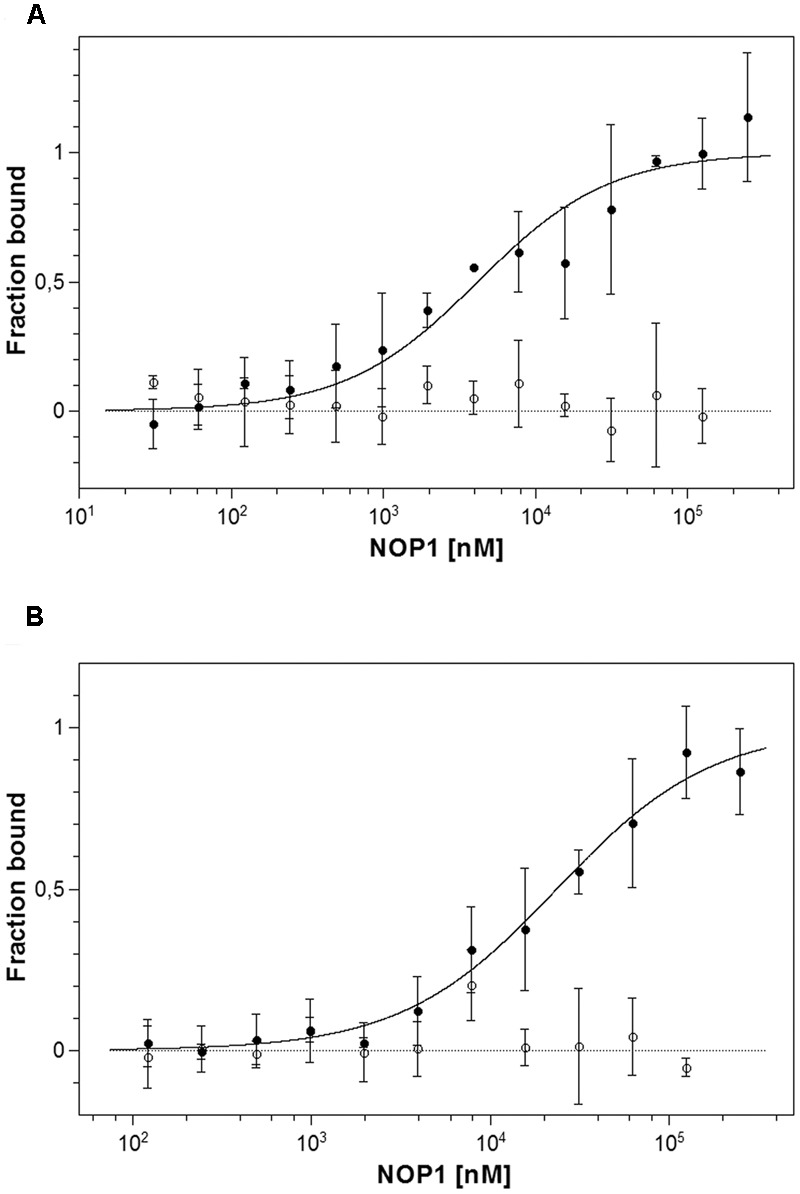
Microscale thermophoresis (MST) interaction studies of tomato ethylene receptors to NOP-1. **(A)** Determination of *K*_d_ value of unlabeled NOP-1 peptide to LeETR4 (bbb) based on MST is shown. A *K*_d_ value of 4.15 ± 0.85 μM was obtained. A negative control using chemically denatured LeETR4 (○) shows no further interaction with binding partner NOP-1. **(B)** Calculation of *K*_d_ value of the small peptide NOP-1 to NR (bbb) using MST technology resulted in a *K*_d_ value of 23.52 ± 1.99 μM. Chemically denatured NR (○) indicates no further binding event with NOP-1. All data represent the mean of three independent measurements ± standard deviation.

Analysis of protein–ligand interactions by MST was used to monitor and to quantify the interaction of synthetic NOP-1 octapeptide with purified recombinant LeETR4 and NR, respectively. Ligand binding and the related dissociation constant with the isolated receptors were deduced from changes in thermophoresis upon addition of the NOP-1 octapeptide (**Figure 4**). In analogy to previous studies on receptors from *Arabidopsis* and constitutively expressed tomato receptor LeETR1 (Bisson et al., 2016) clear changes of the thermophoretic signal were observed upon addition of the synthetic peptide with purified recombinant LeETR4 and NR receptors that are expressed at high levels at fruit ripening. Selectivity of the peptide–receptor interaction was probed in MST studies with chemically denatured receptor proteins. In these experiments, no change in thermophoresis was detected upon addition of NOP-1 (**Figure 4**). The apparent dissociation constant (*K*_d_) calculated from the changes in thermophoresis induced by different amounts of NOP-1 added to fluorescently labeled tomato receptors was 4.15 ± 0.85 μM for LeETR4 and 23.52 ± 1.99 μM for NR, respectively (**Figure 4**). Both numbers are in the lower micromolar range and together with the negative controls on denatured receptor proteins are indicative of efficient and specific binding of NOP-1 to tomato receptors LeETR4 and NR.

### Impact of NOP-1 on Fruit Ripening and Fruit Quality

**FIGURE 5:**
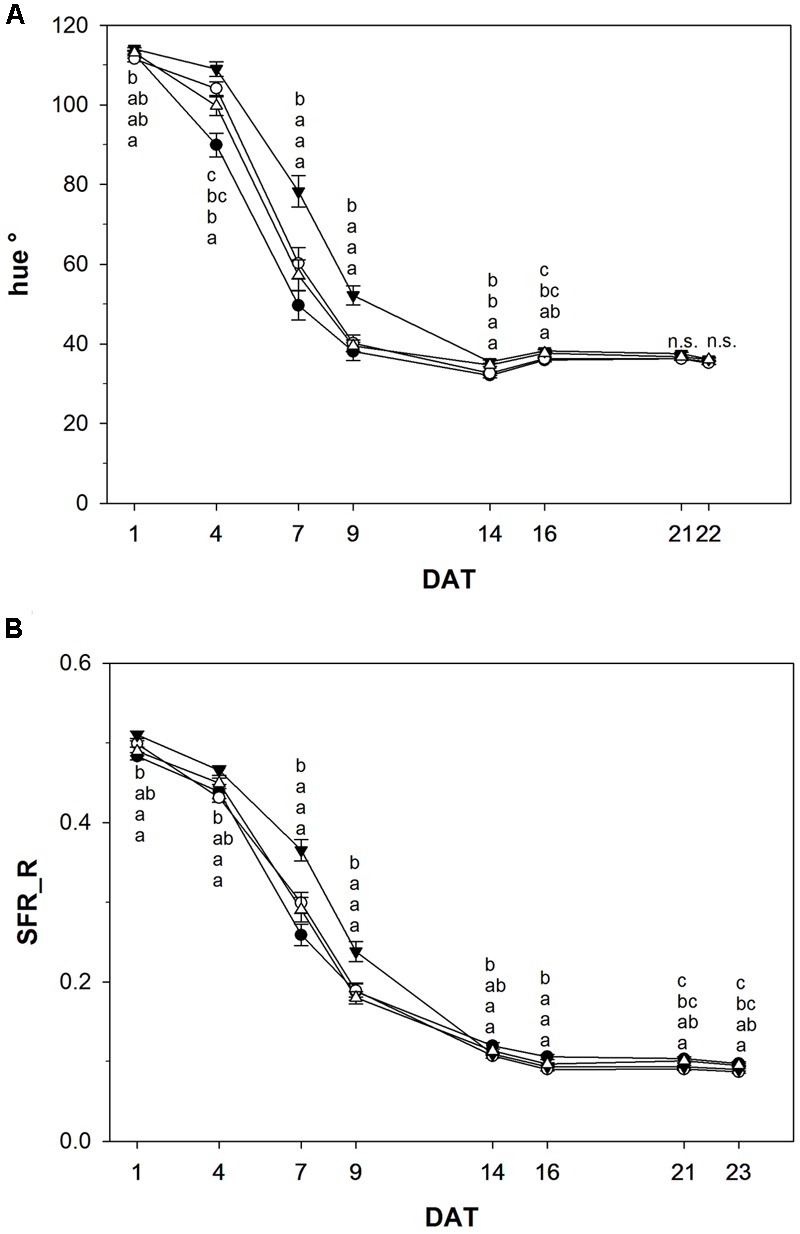
Hue° index **(A)** and SFR_R index **(B)** of control tomato fruits (bbb) and tomato fruits treated with NOP-1 400 μM (○), 1000 μM (ccc) or 2000 μM (Δ). Data are means ± SE, *n* = 3 × 25, – 5 each week, different letters indicate significant differences between treatments on each measurement day (Tukey’s HSD, α ≤ 0.05).

For many fruits and vegetables color development is the most important external characteristic to assess ripeness and post-harvest life. Color change from green to red was slowed down in tomato fruits treated with NOP-1 (1000 μM), as indicated by the hue° index which was significantly higher on DAT 4, 7, and 9 in this treatment group as compared to control (untreated) fruits (**Figure 5A**). For treatment with 400 or 2000 μM NOP-1 slower color change as compared to control fruits were observed only at DAT 4, and to a smaller extent at DAT 7. Thereafter, color change of treated fruits was similar to controls. From DAT 14 onwards, there were no significant differences in hue° among all evaluated treatments. Similar to the observed effects on the hue index, application of 1000 μM NOP-1 also resulted in a significantly higher Simple Fluorescence Index (SFR_R, estimating chlorophyll concentration) on DAT 4, 7, and 9 (**Figure 5B**). A slight increase in SFR_R compared to non-treated fruits was further observed for fruits treated with 400 and 2000 μM NOP-1 at DAT 7. Finally, all treatments reached similar values on DAT 14 and no further changes were observed until the end of the experiment (DAT 23).

**FIGURE 6:**
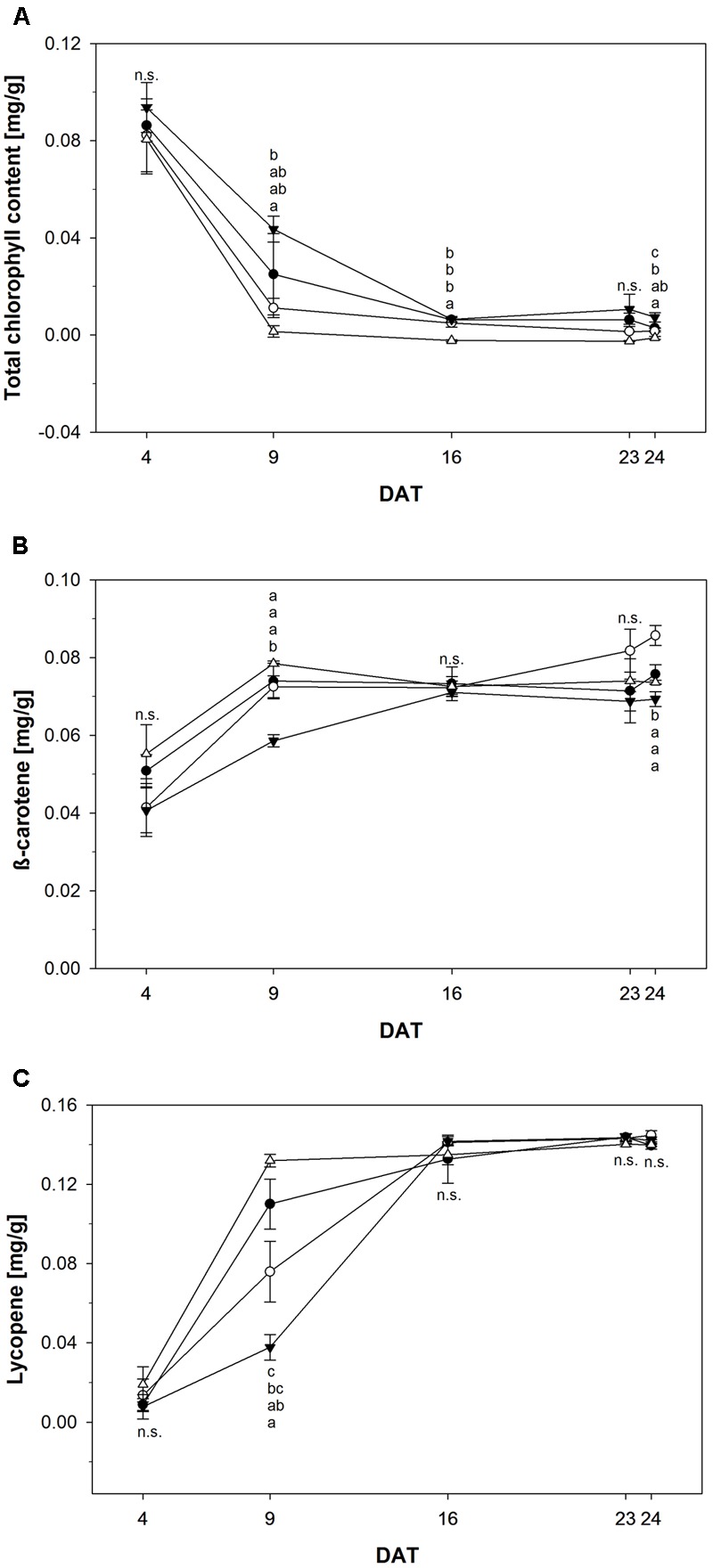
**(A)** Total chlorophyll content of control tomato fruits (bbb) and tomato fruits treated with NOP-1 400 μM (○), 1000 μM (ccc) or 2000 μM (Δ). Concentration of β-carotene **(B)** and lycopene **(C)** of control tomato fruits (bbb) and tomato fruits treated with NOP-1 400 μM (○), 1000 μM (ccc) or 2000 μM (Δ). Data are means ± SE, *n* = 5, different letters indicate significant differences between treatments on each measurement day (Tukey’s HSD, α ≤ 0.05).

Color development is another sensitive but invasive measure to monitor ripening. The chlorophyll content of tomato fruits over time is shown in **Figure 6A**. Fruits treated with 1000 μM NOP-1 showed higher concentrations of total chlorophyll compared to non-treated controls throughout the experiment. While total chlorophyll decreased rapidly to about 30% after DAT 9 in controls, only 50% of the pigment originally present was degraded in fruits treated with 1000 μM at this time. However, over time chlorophyll breakdown in fruits treated with 1000 μM NOP-1 converged to chlorophyll degradation in controls and had essentially ceased after DAT 16. Surprisingly, fruits treated with 400 and 2000 μM NOP-1 showed a more pronounced chlorophyll breakdown on DAT 9 than non-treated controls. However, with further progression of the experiment chlorophyll degradation in these fruits ceased and chlorophyll levels adapted to controls on DAT 16–24.

In contrast to the observed degradation in chlorophyll concentration of β-carotene increased in all treatments throughout the experiment (**Figure 6B**). However, fruits treated with 1000 μM NOP-1 showed a slower increase in β-carotene concentration and revealed significantly lower levels of this pigment at DAT 9 when compared to all other treatments which essentially showed the same pattern for the increase of this carotenoid during ripening. Over time β-carotene in the fruits treated with 1000 μM NOP-1 increased to control levels. All treatments showed comparable levels of this carotene on DAT 16 and concentration of this pigment remained constant throughout the further experiment. Slightly higher concentrations of β-carotene were observed for the 400 μM treatment at DAT 23–24.

**FIGURE 7:**
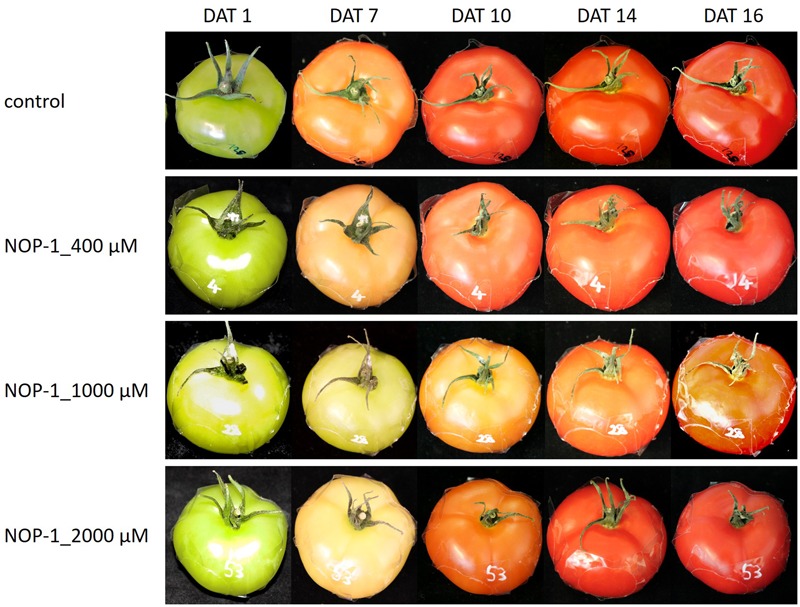
Visual images of fruits treated at different concentrations of NOP-1. Representative photos of whole fruits treated with 400, 1000 and 2000 μM NOP-1 on DAT 1, 7, 10, 14, and 16. Control fruits are depicted in the upper row.

The concentration of lycopene principally responsible for the characteristic deep-red color of ripe tomato fruits, increased significantly in all treatment groups (**Figure 6C**). Similar to the pattern observed for β-carotene, treatment with 1000 μM NOP-1 showed the largest delay in pigment accumulation at DAT 9 where only 33% of the lycopene level of non-treated controls was measured. Treatment with 400 μM of the synthetic peptide still resulted in a delay of lycopene accumulation of about 69% compared to non-treated controls. According to the pattern observed with β-carotene, lycopene levels in all treatments adjusted to similar concentrations after DAT 16 and stayed constant for the rest of the experiment. Highest concentrations of lycopene were measured for all treatments in completely ripe tomatoes at DAT 24. Total color development of fruits treated at different concentrations of NOP-1 is illustrated by visual images of whole fruits (**Figure 7**).

**FIGURE 8:**
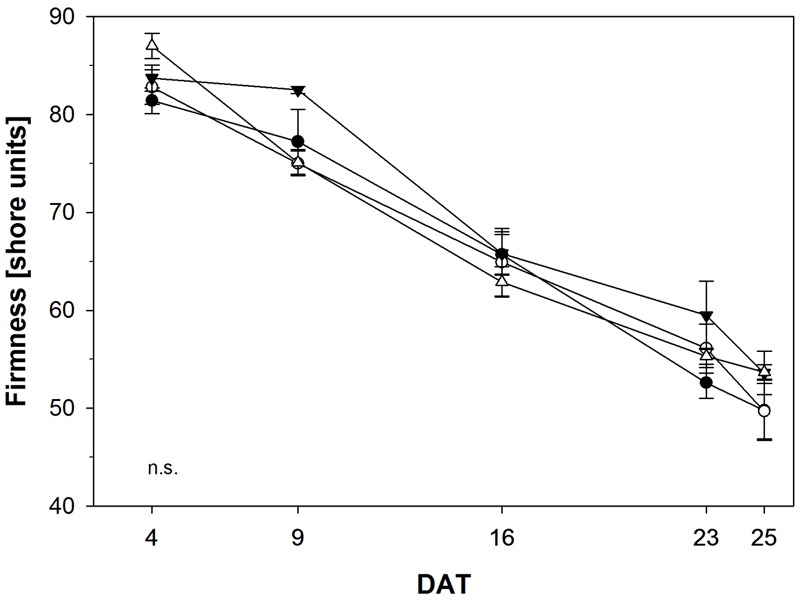
Fruit firmness of control tomato fruits (bbb) and tomato fruits treated with NOP-1 400 μM (○), 1000 μM (ccc) or 2000 μM (Δ). Data are means ± SE, *n* = 5. Statistical analysis according to Tukey’s HSD (α ≤ 0.05) revealed no significant (n.s.) difference in all treatments.

Fruit soften during ripening due to biochemical processes resulting in the breakdown of cell-wall polymers. Hence, firmness is an indirect measurement of ripeness and represents one of the most important variables for fruit quality. Consequently, we determined fruit firmness in tomato fruits treated with NOP-1 and non-treated controls using a non-destructive sensor and a shore scale ranging from 0 to 100 units. Firmness decreased from 80–89 shore to 55–50 shore in the course of the experiment (**Figure 8**). Firmest fruits were observed at the beginning of the experiment, softest fruits were measured at the end. Control, treatment with NOP-1 at concentrations of 400 and 2000 μM showed a continuous decrease in fruit firmness over time, whereas firmness was unaffected at early stages (DAT 4–9) in fruits treated with 1000 μM NOP-1. However, after the initial lag phase firmness decreased to similar levels in these fruits as observed for the other treatments on DAT 16 and later stages. All treatments showed similar numbers for fruit firmness and thereby comparable fruit quality at the end of the experiment on DAT 25.

## Discussion

Previous studies on *Arabidopsis* demonstrated that the small basic peptide NOP-1 derived from the natural NLS-sequence of the ethylene regulator protein EIN2 is able to disrupt ethylene signaling and inhibit plant ethylene responses. Protein–protein interaction studies on recombinant purified proteins EIN2 and ETR1 and related FRET studies in planta suggest that the inhibitory peptide competes for binding of EIN2 at the receptors offering a novel way to interfere with ethylene signal transduction and ethylene responses in planta (Bisson and Groth, 2015; Bisson et al., 2016). The high conservation of the NLS-motif among the plant kingdom (see Supplementary Figure S1 in Bisson et al., 2016) and the level of homology in ethylene receptors open up new avenues for ripening control of fruits and vegetables by biological peptides in modern agriculture and horticulture. Initial studies on tomato, a climacteric fruit of high economic and nutritional impact serving as model to study fruit ripening, support these ideas and confirm the results obtained with the *Arabidopsis* genetic model.

In order to further evaluate the potential of the inhibitory peptide identified in previous studies, we have further analyzed molecular and physiological effects of the basic NLS-derived peptide NOP-1 on tomato. Our studies with purified recombinant receptors LeETR4 and NR, which are both highly expressed in ripening fruit, reveal efficient binding of the peptide to both receptors and thereby confirm that the NOP-1 octapeptide may interact with receptors from both receptor subfamilies. Both receptors interact with the peptide at affinities in the lower μM-range, but interaction with LeETR4 seems to be stronger.

Noteworthy, previous studies demonstrate an even stronger binding affinity of NOP-1 at tomato receptor LeETR1 – a receptor of subfamily I continuously expressed throughout the plant. However, LeETR4 and NR are quite different from LeETR1 with respect to kinase activity as well as in the number and their type of phosphorylation sites (Kamiyoshihara et al., 2012). Keeping further in mind the actual sequence identities of *Arabidopsis* ETR1 and tomato LeETR1/NR/LeETR4 of 81, 69, and 41%, the observed range in affinities of different receptors and receptor subfamilies for NOP-1 is not surprising at all. Bearing in mind that NOP-1 was derived from the NLS motif in EIN2 the different binding affinities observed with the peptide may also suggest that receptors have different affinities for the EIN2 central hub.

In associated post-harvest studies, we have evaluated changes in color and texture development of tomato fruits treated with different concentrations of NOP-1 and the impact of the NLS-derived peptide on fruit ripening and fruit quality. Our studies show clear effects on fruit ripening at concentrations of 1000 μM, whereas effects on color development at 400 μM are substantially less pronounced and manifest only at early ripening stages (DAT 9 – see **Figure 6C**). In total, a concentration of 400 μM NOP-1 applied to the fruit surface as microdroplets seems to be too low for significant inhibition of the ethylene transduction cascade, whereas 1000 μM allows a ripening delay, as expressed by the chlorophyll degradation and lycopene and β-carotene accumulation (**Figure 6**). When applied at 2000 μM concentration no effect of NOP-1 was observed which might be related to concentration-dependent aggregation or changes in secondary structure (Garg et al., 2013), which may impair uptake of the peptide by the fruit surface. Alternatively, the fact that no significant positive effect on ripening delay was observed at 2000 μM concentration in contrast to 1000 μM NOP-1 may be explained by differences in droplet–surface–uptake interactions at the different concentrations. In this case, the solution concentration of 1000 μM showed higher uptake of NOP-1 at the same contact surface area (i.e., tomato fruit surfaces), possibly due to the optimum dose/concentration/interfacial area which apparently causes the maximum penetration. For 2000 μM droplet the NOP-1 loading was twice as high at the same water volume. Consequently, this may have reduced the droplet viscosity on the cuticle, also apparent by its modified macroscopic appearance and altered biomechanical properties (Domínguez et al., 2011; Burkhardt and Hunsche, 2013). In consequence, penetration and uptake of NOP1 may have been reduced, resulting in a less effective 2000 μM treatment.

Improving shelf life and nutritional quality of tomato fruits is difficult to achieve with the methods currently in use. Maintaining adequate storage conditions is expensive and might cause chilling injury if used improperly (Passam et al., 2007). Even though genetic modifications reducing gene expression of proteins involved in ethylene synthesis are possible in principle (Abano and Buah, 2015) these procedures are banned by law throughout Europe and have low acceptance among European consumers. Chemical methods such as application of AVG to inhibit ethylene biosynthesis or treatment with 1-MCP to inhibit ethylene response significantly delay ripening and slow down lycopene synthesis and chlorophyll breakdown (Saltveit, 2005; Passam et al., 2007). The drawbacks of these methods correlating with the restricted use of both chemicals on tomatoes in Europe also relate to quality losses in taste development or to complete arrest in maturation, as observed in some cases (Passam et al., 2007).

In summary, our study shows that the NOP-1 octapeptide derived from the NLS-motif at the EIN2 C-terminus is a potent inhibitor of the maturation process in tomato. The peptide efficiently binds to different receptor isoforms and, when applied to the surface of immature fruit, successfully delays the ripening process without impairment of final overall fruit quality at the fully mature stage. This novel approach to delay fruit ripening is making use of a synthetic peptide that corresponds to the highly conserved NLS-motif in all known EIN2 sequences and holds great promise to control processes such as ripening or senescence in horticultural and agricultural applications.

## Author Contributions

GG conceived the project. GG, GN, and MH planned, designed and supervised the research. MK, SK, and LM performed the experiments and contributed equally to this work. All authors contributed to data analysis and the writing of the manuscript.

## Conflict of Interest Statement

MH is associate professor at the University of Bonn and Global Head of R&D of the company COMPO EXPERT GmbH. The other authors declare that the research was conducted in the absence of any commercial or financial relationships that could be construed as a potential conflict of interest.

## Abbreviations

ACC: 1-aminocyclopropane-1-carboxylic acid
LeETR: ethylene receptor from tomato
NLS: Nuclear localization signal
NR: NeverRipe
